# Supplementary material for: Cost-effectiveness and benefit-cost analyses of promoting handwashing with soap: A systematic review
Source: PLoS Med. 2026 Apr 3;23(4):e1004982. doi: 10.1371/journal.pmed.1004982 (PMC13065014; doi:10.1371/journal.pmed.1004982)
Supplement: S1 Text — (DOCX) [file pmed.1004982.s002.docx]

**S1 Text. Search strategy**

As applied on OVID-SP interface (Medline, EMBASE, Global Health, Econlit)

|  | | HANDWASHING WITH SOAP |
| --- | --- | --- |
| 1 | | (handwash* or (hand* adj3 (wash* or hygiene))).mp |
| 2 | | (hand* adj3 soap*).mp |
| 3 | | ((hygiene or handwash*) adj3 (promot* or educat* or behavio*)).mp |
| 4 | | 1 or 2 or 3 |
|  | | ECONOMIC EVALUATIONS |
| 5 | (cost-benefit or benefit-cost or cost-effectiv* or cost-utility or (econ* adj1 eval*) or (cost adj2 efficien*) or value for money or value-for-money).mp |  |
|  | | COMBINING ALL TERMS |
| 6 | | 4 and 5 |
|  | | LIMITS |
| 7 | limit 6 to yr="1980 -Current" |  |
| 8 | | Limit 7 to (humans) |

As applied on Web of Science

|  | HANDWASHING WITH SOAP |
| --- | --- |
| # 1 | TS=(handwash* or (hand* NEAR/3 (wash* or hygiene) )) |
| # 2 | TS=(hand* NEAR/3 soap*) |
| # 3 | TS= ((hygiene or handwash*) NEAR/3 (promot* or educat* or behavio*) ) |
| # 4 | #3 OR #2 OR #1 |
|  | ECONOMIC EVALUATIONS |
| # 5 | TS= (cost-benefit or benefit-cost or cost-effectiv* or cost-utility or (econ* NEAR/1 eval*) or (cost NEAR/2 efficien*) or value for money or value-for-money) |
|  | COMBINING ALL TERMS |
| # 6 | #4 AND #6 |

We also searched the below databases and websites, adapting the above-listed search terms to their interfaces:

- Cochrane library,
- International Bibliography of the Social Sciences,
- Global Health Cost Effectiveness Analysis Registry,
- NHS Economic Evaluation Database
- International bibliography of the social sciences
- National Bureau of Economic Research
- International Initiative for Impact Evaluation
- Research Papers in Economics,
- WHO Index Medicus (all regions)
- Copenhagen Consensus Centre
