# Supplementary material for: Cost-effectiveness and benefit-cost analyses of promoting handwashing with soap: A systematic review
Source: PLoS Med. 2026 Apr 3;23(4):e1004982. doi: 10.1371/journal.pmed.1004982 (PMC13065014; doi:10.1371/journal.pmed.1004982)
Supplement: S1 Table — We included studies of combined interventions if they reported effect estimates separately for the handwashing component or clearly had handwashing as a “major” component. (DOCX) [file pmed.1004982.s003.docx]

**S1 Table. Categories of the extent of combination of hygiene interventions**

|  | **Descriptor** |
| --- | --- |
| **1. Handwashing only** | Interventions focused on handwashing alone (or alongside other body washing, e.g. bathing, or face-washing) |
| **2. Handwashing majority (≥50% of messages)** | Interventions focused on handwashing alongside other components, but where the handwashing component was the behavioural target in ≥50% of intervention messages. No distinction is made regarding what handwashing is combined with (e.g. household water treatment, food hygiene). |
| **3. Handwashing minority (~25-50% of messages)** | Interventions where handwashing is a major part of a broad intervention, but is the behavioural target in <50% of intervention messages. To be included, the CEA/BCA itself (rather than a referenced paper, e.g. process evaluation), must indicate that handwashing is part of the intervention anywhere in the methods section. In cases where “hygiene” is mentioned rather than handwashing, but references and other details (e.g. soap) imply handwashing, this is interpreted as handwashing. |
