# Supplementary material for: Cost-effectiveness and benefit-cost analyses of promoting handwashing with soap: A systematic review
Source: PLoS Med. 2026 Apr 3;23(4):e1004982. doi: 10.1371/journal.pmed.1004982 (PMC13065014; doi:10.1371/journal.pmed.1004982)
Supplement: S2 Table — (DOCX) [file pmed.1004982.s004.docx]

**S2 Table. Scoring for CHEERS**

| **Area** | **Reporting item (from Husereau et al., 2013)** | | **Interpretation for scoring in this study (authors)** | | |
| --- | --- | --- | --- | --- | --- |
| **Framing** | **1. Title** | Identifies the study as an EE or uses specific terms such as CEA, and describes the interventions compared. | 1 - includes, CEA, BCA, EE, or similar, in the title | 0.5 - implication of EE but not with clear terms | 0 - nothing implies EE |
|  | **2. Abstract** | Structured summary of objectives, perspective, setting, methods, results (including base case & uncertainty) | 1 - abstract contains most of these | 0.5 - abstract provides some of above, but missing some of the most important (intervention, methods, results) | 0 - abstract provides very few of these |
|  | **3. Background and objectives** | Provides broader context for the study. Presents the study question and its relevance for policy or practice decisions. | 1 - study aim/question presented with relevance for decisions | 0.5 - EITHER clear aim but limited context OR good relevance re decisions, but unclear aim or question regarding EE | 0 - BOTH aim/question AND relevance for decisions unclear |
| **Population, setting** | **4. Target population and subgroups** | Describes characteristics of the base case population and subgroups analysed, including why they were chosen. | 1 - study population sufficiently described, including any assumptions | 0.5 - study population described, but too briefly in respect of policy-relevant characteristics | 0 - study population unclear |
|  | **5. Setting and location** | States relevant aspects of the system(s) in which the decision(s) need(s) to be made. | 1 - characteristics of decision context described | 0.5 - decision context described but insufficiently | 0 - decision context appears not to have been considered |
| **Key methods decisions** | **6. Study perspective** | Describes the perspective of the study and relate this to the costs being evaluated. | 1 - perspective stated clearly and interpreted | 0.5 - perspective vaguely stated, but sufficient detail such that it can easily be discerned | 0 - perspective unclear |
|  | **7. Comparators** | Describes the interventions or strategies being compared and state why they were chosen. | 1 - intervention and comparator both well-described | 0.5 - limited detail on comparator, but doesn’t substantially harm interpretation | 0 - lack of detail on comparator limits interpretation |
|  | **8. Time horizon** | States the time horizon(s) over which costs and consequences are being evaluated and say why appropriate. | 1 - time horizon stated and explained | 0.5 - time horizon stated without context, or clearly implied | 0 - time horizon unclear, which limits interpretation |
|  | **9. Discount rate** | Reports the choice of discount rate(s) used for costs and outcomes and say why appropriate. | 1 - rate reported and justified | 0.5 - rate reported but unclear how applied or not justified / referenced | 0 - no discounting, or unclear how applied |
| **Outcomes and costs** | **10. Choice of outcomes** | Describes what outcomes were used as the measure(s) of benefit in the evaluation and their relevance for the type of analysis performed. | 1 - outcomes / benefits clear | 0.5 - lack of clarity on some but not all benefits | 0 - outcomes / benefits very unclear |
|  | **11. Measurement of effectiveness** | *(i) Single study:*Describes features of design and why sufficient, (ii) *Synthesis-based*: describes identification & synthesis of included studies | 1 - source of effects data explained, discussed and justified | 0.5 - source and important assumptions clear, but not discussed / justified | 0 - source or important assumptions unclear |
|  | **12. Measurement & valuation of pref.-based outcomes** | If applicable, describe the population and methods used to elicit preferences for outcomes. | 1 - if DALYs used, source of weights referenced / discussed | 0.5 - slightly unclear on weights | 0 - very unclear on weights |
|  | **13. Estimating resources and costs** | Describes approaches used to estimate and value resource use, and any adjustments made to approximate to opportunity costs. | 1 - Approach to costing and sources of data are clear, with quality discussed | 0.5 - some information on costing missing, OR quality of data sources unclear / not discussed | 0 - much information / discussion of costing data sources missing |
|  | **14. Currency, price date, and conversion** | Reports the dates of the estimated resource quantities and unit costs, and methods for adjusting to the year/currency of analysis. | 1 - currency dates and conversions clear | 0.5 - minor issues of clarity | 0 - currency dates OR conversions not reported |
| **Modelling** | **15. Choice of model** | Describes and give reasons for the specific type of decision-analytical model used. | 1 - model is clearly described | 0.5 - model description has some limitations | 0 - model unclear |
|  | **16. Assumptions** | Describes all structural or other assumptions underpinning the decision-analytical model. | 1 - model assumptions clear, allowing reproducibility | 0.5 - most model assumptions clear, but not reproducible | 0 - model assumptions unclear |
|  | **17. Analytical methods** | Describes all analytical methods supporting the evaluation (e.g. missing or censored data; population heterogeneity) | 1 - explains important steps, e.g. how cost categories / types summed, dealt with missing data / outliers | 0.5 - partially explained | 0 - poorly explained |
|  | **18. Study parameters** | Report the values, ranges, references (and, if used, probability distributions) for all parameters. | 1 - values, ranges and references for input parameters reported | 0.5 - some model inputs described, but not values, ranges, references | 0 - input parameters poorly described |
| **Results and sensitivity** | **19. Incremental costs and outcomes** | Reports mean values for the main categories of estimated costs and outcomes, as well as mean differences between comparator groups | 1 - mean values for cost categories / outcomes reported | 0.5 - costs/outcomes only reported in aggregate, not disaggregated by category | 0 - very unclear reporting of costs / outcomes |
|  | **20. Characterising uncertainty** | Describes the effects of sampling and/or model uncertainty, with impact of methodological assumptions (e.g. discount rate) | 1 - parameter and structural uncertainty adequately characterised | 0.5 - only parameter OR structural uncertainty adequately characterised | 0 - limited characterisation of sources of uncertainty |
|  | **21. Characterising heterogeneity** | If applicable, reports differences in costs, outcomes, or cost-effectiveness that can be explained by variations between subgroups. | 1 - if sub-group analysis conducted, it is clear | 0.5 - if sub-group analysis conducted, it is fairly clear | 0 - if sub-group analysis conducted, it is unclear |
| **Other** | **22. Findings, limitations, generalisability** | Summarises key study findings and limitations, describing how results support the conclusions, and their generalisability. | 1 - findings / conclusions clearly linked and limitations discussed | 0.5 - conclusions unclear or not linked to findings, OR limitations not discussed | 0 - conclusions and limitations unclear |
|  | **23. Source of funding** | Describes how the study was funded and the role of the funder in the identification, design, conduct, and reporting of the analysis. | 1 - funder noted | n/a | . - funder unclear (not a norm) |
|  | **24. Conflicts of interest** | Describes any potential for conflict of interest of study contributors in accordance with journal policy. | 1 - COI adequately described | n/a | . - COI not described (not a norm) |
