# Supplementary material for: Cost-effectiveness and benefit-cost analyses of promoting handwashing with soap: A systematic review
Source: PLoS Med. 2026 Apr 3;23(4):e1004982. doi: 10.1371/journal.pmed.1004982 (PMC13065014; doi:10.1371/journal.pmed.1004982)
Supplement: S3 Table — (DOCX) [file pmed.1004982.s005.docx]

**S3 Table.** **Studies excluded at full-text review with reasons for exclusion**

| **First author** | **Year** | **Title** | **Journal** | **Primary reason for exclusion** | **Comment** |
| --- | --- | --- | --- | --- | --- |
| Xiao | 1997 | Evaluation of effectiveness of comprehensive control for diarrhoea diseases in rural areas of east Fujian and analysis of its cost-benefit | Chinese Journal of Preventive Medicine | wrong intervention (HW <25% of content) | Broad intervention including water supply, sanitation, road renovation, ditch cleaning, health education (boil water, handwashing, fly prevention), advice to farmers (fences for poultry / livestock) |
| Uhari | 1999 | An open randomized controlled trial of infection prevention in child day-care centers. | Pediatric Infectious Disease Journal | not an economic evaluation | Does not report cost-effectiveness estimates, nor even costs |
| Guinan | 2002 | The effect of a comprehensive handwashing program on absenteeism in elementary schools | American Journal of Infection Control | partial economic evaluation | Measures cost savings only, does not value benefits |
| Mascie-Taylor | 2003 | The cost-effectiveness of health education in improving knowledge and awareness about intestinal parasites in rural Bangladesh | Economics and Human Biology | another reporting of an included study | Duplicates methods/results of Mascie-Taylor 1999 |
| Cimiotti | 2004 | A cost comparison of hand hygiene regimens | Nursing economics | wrong setting (healthcare, workplace, etc.) | Healthcare setting (but also reports costs not cost-effectiveness) |
| Hutton | 2007 | Global cost-benefit analysis of water supply and sanitation interventions | Journal of Water and Health | wrong intervention (0% or unclear HW) | listed interventions do not include hygiene/handwashing |
| Haller | 2007 | Estimating the costs and health benefits of water and sanitation improvements at global level | Journal of Water and Health | wrong intervention (0% or unclear HW) | listed interventions do not include hygiene/handwashing |
| Renwick | 2007 | Cost -Benefit Analysis of National and Regional Integrated Biogas and Sanitation Programs in Sub -Saharan Africa | Discussion paper | investment case for planned programme | Planning for a specific biogas, sanitation and hygiene programme |
| Edmond | 2010 | New Approaches to Preventing, Diagnosing, and Treating Neonatal Sepsis | PLoS Medicine | not an economic evaluation | Review article |
| Graves | 2012 | Evaluating the economics of the Australian National Hand Hygiene Initiative | Healthcare Infection | wrong setting (healthcare, workplace, etc.) | Healthcare setting (but also reports costs not cost-effectiveness) |
| Chen | 2013 | Cost-effectiveness of influenza control measures: A dynamic transmission model-based analysis | Epidemiology and Infection | partial economic evaluation | Estimates unit cost per person per year, not cost-effectiveness |
| Graves | 2013 | Linking scientific evidence and decision making: A case study of hand hygiene interventions | Infection Control and Hospital Epidemiology | wrong setting (healthcare, workplace, etc.) | Healthcare setting (but also reports costs not cost-effectiveness) |
| Zhang | 2013 | Promoting clean hands among children in Uganda: A school-based intervention using 'tippy-taps' | Public Health | partial economic evaluation | Reports costs only |
| Ataniya-zova | 2014 | A Cost-Benefit Analysis of Early Childhood Hygiene Interventions in Uzbekistan | Eurasian Journal of Business and Economics | partial economic evaluation | Measures cost savings only, does not value benefits as per our definition of benefit-cost analysis |
| Khazeni | 2014 | Health and Economic Benefits of Early Vaccination and Nonpharmaceutical Interventions for a Human Influenza A (H7N9) Pandemic | Annals of Internal Medicine | wrong intervention (HW <25% of content) | Intervention is closures of schools and child care facilities; home isolation; cough etiquette; hand washing; use of alcohol-based hand gels; and facemasks. Also unclear whether it is specifically promotion of handwashing or merely the practice of handwashing |
| Whinnery | 2016 | Handwashing with a water-efficient tap and low-cost foaming soap: the Povu Poa "Cool Foam" system in Kenya. | Global Health: Science and Practice | partial economic evaluation | Reports costs only |
| Cunning-ham | 2017 | Community video: An adaptable and effective tool for nutrition social and behavior change | Annals of Nutrition and Metabolism | not a study (conference abstract, opinion, etc.) | Conference abstract |
| OECD | 2018 | Stemming the Superbug Tide: Just A Few Dollars More | OECD report | wrong setting (healthcare, workplace, etc.) | Healthcare setting |
| Senapati | 2019 | A cholera metapopulation model interlinking migration with intervention strategies - a case study of zimbabwe (2008-2009) | Journal of Biological systems | another reporting of an included study | Duplicates methods/results of Sardar 2013 |
| Asamoah | 2020 | Global stability and cost-effectiveness analysis of COVID-19 considering the impact of the environment: using data from Ghana | Chaos Solitons & Fractals | wrong intervention (not promotion or provision) | Intervention is the practice of "cover coughs/ sneezes, wash hands after coughs/sneezes" rather than the promotion of that practice, per the description but also the costs. |
| Bagepally | 2021 | Cost-effectiveness of surgical mask, N-95 respirator, hand-hygiene and surgical mask with hand hygiene in the prevention of COVID-19: Cost effectiveness analysis from Indian context | Clinical Epidemiology and Global Health | wrong intervention (not promotion or provision) | Intervention is the practice of handwashing rather than the promotion of that practice, and costs only include "hand wash"/sanitiser |
| Zafari | 2021 | The cost-effectiveness of common strategies for the prevention of transmission of SARSCoV- 2 in universities | PLoS ONE | wrong intervention (not promotion or provision) | Intervention is the practice of handwashing rather than the promotion of that practice. In any case, handwashing and masking was the comparator ("status quo arm") rather than an intervention. |
| Asamoah | 2021 | Sensitivity assessment and optimal economic evaluation of a new COVID-19 compartmental epidemic model with control interventions | Chaos, Solitons and Fractals | wrong intervention (HW <25% of content) | Intervention is physical distancing, media advocacy, wearing of a nose mask, the use of hand sanitiser-washing of hands, lockdowns, stringent safety measures in hospitals (and/or isolation centres), with a constant supply of effective personal protective equipment (PPE)), testing-diagnoses and contact tracing. So it is also unclear if soap or sanitisier, and the intervention is practice rather than promotion. |
| Reddy | 2021 | Cost-effectiveness of public health strategies for COVID-19 epidemic control in South Africa: a microsimulation modelling study | Lancet Global Health | wrong intervention (not promotion or provision) | Handwashing is not one of the strategies tested |
| Wang | 2022 | Cost-Effectiveness of Public Health Measures to Control COVID-19 in China: A Microsimulation Modeling Study | Frontiers in Public Health | wrong intervention (not promotion or provision) | Intervention is the practice of handwashing rather than the promotion of that practice, per the description but also the included costs. |
| Akinyemi | 2023 | A tale of two countries: Optimal control and cost-effectiveness analysis of monkeypox disease in Germany and Nigeria | Healthcare Analytics | wrong intervention (not promotion or provision) | Intervention is "Behavioral modification and personal hygiene" and involves practice of handwashing rather than its promotion. |
| Edward | 2024 | On the role of vaccination, health education, and hygiene compliance in the elimination and control of Hepatitis A Virus: An optimal control approach | Informatics in Medicine Unlocked | wrong intervention (not promotion or provision) | Not clear what "hygiene" is and whether it includes handwashing, nor what is included in "health education". No cost information provided so unclear whether handwashing is part of it or not. |
| Omura | 2024 | Promoting healthy practices among schools and children in rural bangladesh: a randomised controlled trial of skill-based health education | BMC public health | partial economic evaluation | Cost study only because it is cost per 0.1 SD increase in the average treatment effect, where outcomes are behaviour (not health or other valued outcome) |
| Ssemanda | 2025 | Cost-effectiveness of interventions toward improving microbial food safety of chicken meat along supply chains in Burkina Faso and Ethiopia | International Journal of Food Microbiology | wrong intervention (not promotion or provision) | Cost itemisation in Supplementary Material makes it clear there is no promotion (Ethiopia study is in homes not workplace) |
| Engida | 2025 | Analysis of a Comprehensive Mathematical Model for Leptospirosis Dynamics: An Optimal Control Application | JOURNAL OF MATHEMATICS | wrong intervention (not promotion or provision) | Nothing about the control option suggests that handwashing is being promoted and no cost details provided to allow understanding of that. |

Abbreviations: HW = handwashing
