# Supplementary material for: Cost-effectiveness and benefit-cost analyses of promoting handwashing with soap: A systematic review
Source: PLoS Med. 2026 Apr 3;23(4):e1004982. doi: 10.1371/journal.pmed.1004982 (PMC13065014; doi:10.1371/journal.pmed.1004982)
Supplement: S4 Table — (DOCX) [file pmed.1004982.s006.docx]

**S4 Table. Further characteristics of included studies**

| **Reference** | **Sensitivity analysis** | **Perspective** | **Comparator and behaviour** | **CHEERS**  **score (%)** |
| --- | --- | --- | --- | --- |
| Varley (1998) | one-way DSA on 1 parameter | Provider (but unclear who is bearing operational costs) | No intervention (unclear HW behaviour) | 66% |
| Mascie-Taylor (1999) | None, but reports results for all children and a subset with high-intensity worm infections | Provider implied (household-borne costs excluded) | No intervention (unclear HW behaviour) | 55% |
| Borghi (2002) | one-way DSA on 8 parameters | Societal (but provider and household perspectives also reported) | No intervention (“before” scenario behaviour) | 79% |
| Larsen (2003) | None | Societal implied (soap/water costs included) | Unclear – appears no intervention (unclear HW behaviour) | 40% |
| Cairncross (2006) | None | Provider implied (facility/soap/water costs excluded) | Unclear – appears no intervention (unclear HW behaviour) | 45% |
| Hansen (2008) | Unclear low/high scenarios for HWWS, plus one-way DSA on few variables for overall package of 65 interventions | Provider (household-borne costs excluded) | Unclear – appears current practice, which is not stated (unclear HW behaviour) | 59% |
| Lachance (2010) | One-way and two-way DSA on many variables, as well as scenario-based DSA | Societal | Thermometer provision only (control arm) but meta-analysis effect estimate used (self-reported HW behaviour) | 90% |
| Machdar (2013) | None | Unclear, appears provider | Unclear – appears no intervention (unclear HW behaviour) | 36% |
| Sardar (2013) | Unclear. Aspires to PSA given the nature of the model but methods are not explained. Distributions of key variables are not provided | Unclear, appears provider | Unclear – appears no intervention (unclear HW behaviour) | 54% |
| Siu (2021) | PSA with cost-effectiveness plane and cost-effectiveness acceptability curve. However, no DSA and not 95% CI for the ICER. | Societal | Promotion of water use in domestic vegetable gardening (control arm), with baseline/control behaviour reported | 82% |
| Azor-Martinez (2021) | Unclear. Claims Bayesian PSA but methods and distributions are not reported | Societal and provider | Usual handwashing practice (control arm), and sanitiser arm. Behaviour not reported. | 93% |
| Beresniak (2023) | DSA for pandemic scenarios reported for all interventions. Additionally PSA for certain interventions only (not HWWS) with unclear methods and distributions | Health system | Unclear – appears no intervention (unclear HW behaviour) | 55% |
| Whittington (2012) | PSA with uniform distributions for key variables, alongside DSA on adoption/adherence variables | Societal implied (default for BCA) | No intervention (uptake clear, but not baseline level of behaviour) | 81% |
| Larsen (2016) | Two-way DSA on discount rate and health valuation methods | Societal implied (default for BCA) | Unclear – appears no intervention (unclear HW behaviour) | 55% |
| Townsend (2017) | None for BCR. A 95% CI for net benefit is reported but methods for this are unclear. | Provider implied (only includes programme cost, not facility/soap/water/) but also includes avoided cost of illness on benefit side which is inconsistent. | Unclear – appears no intervention (assumptions about baseline behaviour reported) | 41% |
