# Supplementary material for: Cost-effectiveness and benefit-cost analyses of promoting handwashing with soap: A systematic review
Source: PLoS Med. 2026 Apr 3;23(4):e1004982. doi: 10.1371/journal.pmed.1004982 (PMC13065014; doi:10.1371/journal.pmed.1004982)
Supplement: S1 Fig — (DOCX) [file pmed.1004982.s007.docx]

**S1 Figure. Incremental cost per DALY averted for handwashing interventions in high- or medium-quality studies, using Pichon-Riviere cost-effectiveness thresholds for QALYs.**

*
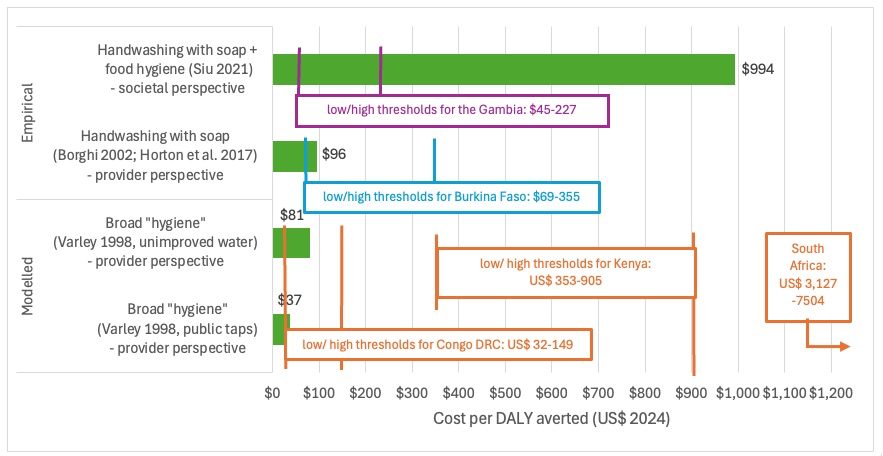
*

Data are presented in US$ 2024, and cost-effectiveness thresholds from those for quality-adjusted life years (QALYs) in Pichon-Riviere et al. (2023). While in theory gaining 1 QALY is equivalent to averting 1 DALY, these measures do have important distinctions in practice. The Ochalek thresholds have been more widely-used for DALY comparisons, which is why we do so in our study. Estimated thresholds in 2024 US$ based on percentages in two different studies applied to GDP per capita are below.

|  | Ochalek et al. (2018) | | Pichon-Riviere et al. (2023) | |
| --- | --- | --- | --- | --- |
|  | low | high | low | high |
| Burkina Faso | 227 | 306 | 69 | 355 |
| The Gambia | 472 | 627 | 45 | 227 |
| Congo DRC | 78 | 97 | 32 | 149 |
| South Africa | 2689 | 3627 | 3127 | 7504 |
| Kenya | 794 | 1037 | 353 | 905 |
