# Supplementary material for: Cost-effectiveness and benefit-cost analyses of promoting handwashing with soap: A systematic review
Source: PLoS Med. 2026 Apr 3;23(4):e1004982. doi: 10.1371/journal.pmed.1004982 (PMC13065014; doi:10.1371/journal.pmed.1004982)
Supplement: S2 Fig — Items are not applicable when norms are not established for that type of publication (e.g., abstract, funding, conflicts of interest) or the study did not use that method (e.g., preference-based outcomes, heterogeneity). (DOCX) [file pmed.1004982.s008.docx]

**S2 Figure. Distribution of CHEERS scores per item**

Note. Items are not applicable when norms are not established for that type of publication (e.g. abstract, funding, conflicts of interest) or the study did not use that method (e.g. preference-based outcomes, heterogeneity).
