# Supplementary figures and images for: Cost-effectiveness and benefit-cost analyses of promoting handwashing with soap: A systematic review
Source: PLoS Med. 2026 Apr 3;23(4):e1004982. doi: 10.1371/journal.pmed.1004982 (PMC13065014; doi:10.1371/journal.pmed.1004982)

**S3 Figure. CHEERS item scores and overall ratings and scores per study**


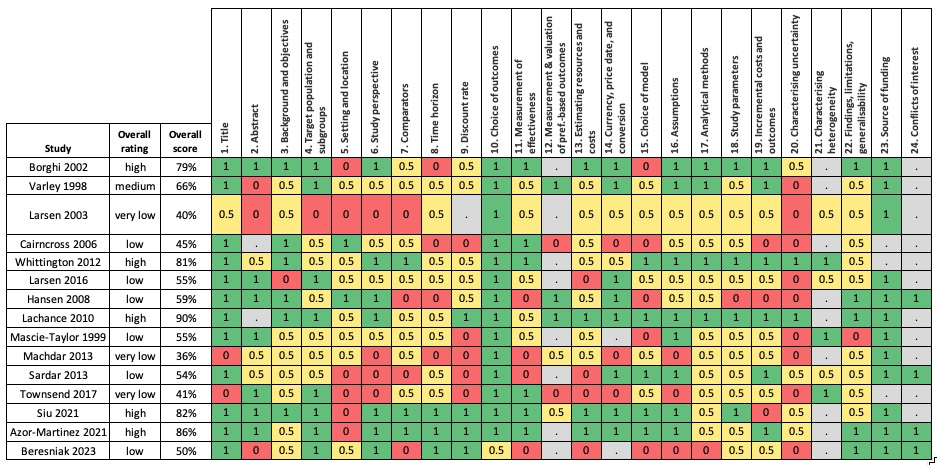

Supplement: S3 Fig — (DOCX) [file pmed.1004982.s009.docx]
