# Supplementary material for: Cost-effectiveness and benefit-cost analyses of promoting handwashing with soap: A systematic review
Source: PLoS Med. 2026 Apr 3;23(4):e1004982. doi: 10.1371/journal.pmed.1004982 (PMC13065014; doi:10.1371/journal.pmed.1004982)
Supplement: S4 Fig — (DOCX) [file pmed.1004982.s010.docx]

**S4 Figure. CHEERS score by year of publication**
